# The Emerging Promise of Pentacyclic Triterpenoid Derivatives as Novel Antiviral Agents Against SARS-CoV-2 Variants

**DOI:** 10.3390/molecules31020325

**Published:** 2026-01-17

**Authors:** Xin Wan, Xiaoxuan Cui, Ke Liang, Junran Huang, Kangan Chen, Wen Chen, Gaopeng Song

**Affiliations:** 1 School of Pharmacy and Laboratory Medicine, Huizhou Health Sciences Polytechnic, Huizhou 516000, China; wanxin0101@126.com (X.W.); cocolk1983@163.com (K.L.); 2 Key Laboratory for Biobased Materials and Energy, Ministry of Education, College of Materials and Energy, South China Agricultural University, Guangzhou 510642, China; 18366523126@163.com (X.C.); 13059137052@163.com (J.H.); cka2293689752@126.com (K.C.)

**Keywords:** SARS-CoV-2, pentacyclic triterpenoids, antiviral activity, structure–activity relationships, mechanism

## Abstract

The continuous emergence of SARS-CoV-2 variants, especially the Omicron strain with its heightened transmissibility, has posed ongoing challenges to the efficacy of existing vaccine and drug regimens. This situation highlights the pressing demand for antiviral drugs employing novel mechanisms of action. Pentacyclic triterpenoids (PTs), a structurally varied group of compounds derived from plants, exhibit both antiviral and anti-inflammatory activities, making them attractive candidates for further therapeutic development. These natural products, along with their saponin derivatives, show broad-spectrum inhibitory effects against multiple SARS-CoV-2 variants (from Alpha to Omicron) via interactions with multiple targets, such as the spike protein, main protease (Mpro), RNA-dependent RNA polymerase (RdRp), and inflammatory signaling pathways. This review consolidates recent findings on PTs and their saponins, emphasizing their influence on the key structural features required for inhibiting viral attachment, membrane fusion, reverse transcription, and protease function. We systematically summarized the structure–activity relationships and their antiviral results of PTs based on different target proteins in existing studies. Furthermore, this work points toward new strategies for designing multi-target PT-based inhibitors with improved efficacy against Omicron and future variants.

## 1. Introduction

Coronaviruses (CoVs), the largest family of RNA viruses, include several members responsible for widespread global infections, notably SARS-CoV, MERS-CoV, and the most recent outbreak of severe acute respiratory syndrome coronavirus 2 (SARS-CoV-2), posing a major public health threat [1,2,3]. The ongoing COVID-19 pandemic, caused by SARS-CoV-2, has severely impacted global health, resulting in over 778 million infections and 7 million deaths [4,5]. During the pandemic, the virus has continuously evolved, generating multiple variants (Alpha, Beta, Delta, Omicron), which present new risks and challenges to national epidemic responses [6,7,8]. To date, the worldwide proliferation of Omicron and its sub-variants has raised significant concerns due to significantly increased transmissibility and further enhanced immune evasion, weakening the effectiveness of vaccines and antibodies acquired from previous infections. Substantial efforts have been exerted to reuse approved anti-SARS-CoV-2 drugs to combat COVID-19. For instance, Remdesivir was the first drug approved for COVID-19 treatment, which can directly target the RNA-dependent RNA polymerase (RdRp) of SARS-CoV-2 to block viral replication [9]. Ritonavir in combination with Nirmatrelvir (marketed as Paxlovid) functions as a specific Mpro inhibitor, blocking the proteolytic cleavage and activation of viral polyproteins to curtail SARS-CoV-2 replication [10]. Moreover, while many new small-molecule inhibitors have been developed and tested in vitro and in vivo, only a small subset of these compounds is currently proceeding to clinical trials, such as Ensitrelvir [11], a 3CLPro inhibitor developed in Japan, and Plitidepsin (Aplidin) [12], a cyclic peptide derived from marine organisms that works by inhibiting eEF1A. Globally, the world continues to grapple with successive waves of COVID-19 triggered by emerging Omicron sub-variants, underscoring the urgency to develop novel, potent inhibitors with innovative mechanisms of action.

**Figure 1:**
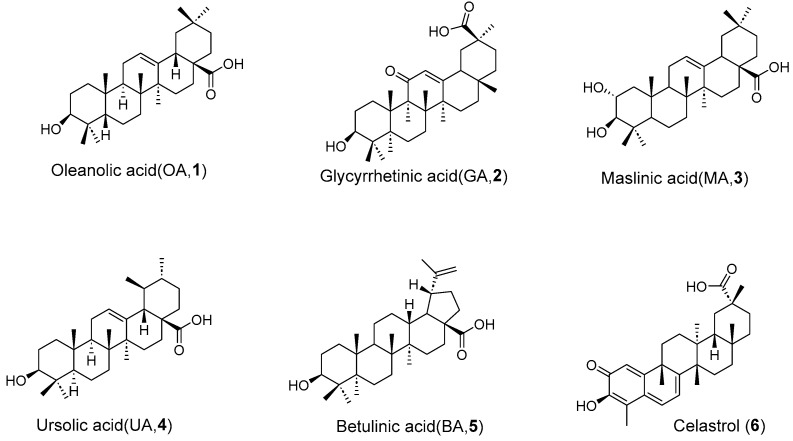
Chemical structures of representative natural PTs **1**–**6**.

Natural products (NPs) are a class of naturally derived chemicals with complex structures and diverse pharmacological activities, which are composed of alkaloids, flavonoids, terpenes, phenylpropanoids, and other members [13]. Within this category, pentacyclic triterpenoids (PTs) emerge as a notable class of plant secondary metabolites, characterized by four six-membered rings (designated A, B, C, D) and a fifth ring (E) that can be either five-membered or six-membered [14]. They can be categorized into four primary types: (a) oleanane-type triterpenes such as oleanolic acid (OA, **1**, Figure 1), glycyrrhetinic acid (GA, **2**, Figure 1), and maslinic acid (MA, **3**, Figure 1), (b) ursane type triterpenes represented by ursolic acid (UA, **4**, Figure 1), (c) lupane type triterpenes including betulin and betulinic acid (BA, **5**, Figure 1), (d) friedelane type triterpenes such as celastrol (**6**, Figure 1) based on the carbon skeleton.

**Figure 2:**
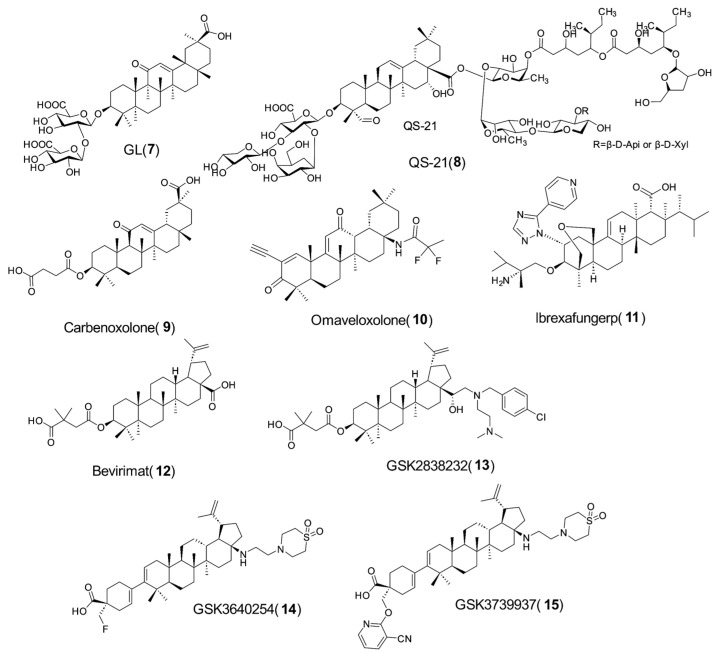
Chemical structures of representative PTs-based drugs **7**–**15** approved in the clinical.

Structurally, PTs feature three typical functional groups: a C-3 hydroxyl group, a 12-ene, and either a C-17 or C-20 carboxylic acid. These structures exhibit distinct characteristics, including molecular rigidity, hydrogen bond donors/acceptors, and a significantly extensive hydrophobic surface area. Such attributes endow PTs with a unique and pivotal role in pharmaceutical research, highlighting their significance in drug discovery. Glycyrrhizinic acid (GL, **7**, Figure 2) and OA have been used in traditional medicine for thousands of years and are now widely used around the world. More recently, several new PTs-based drugs have achieved significant breakthroughs and been used in the clinic, including a complex triterpene glycoside vaccine adjuvant QS-21 (mild local/systemic reactions, no severe adverse events (SAEs)) [15] (**8**, Figure 2), carbenoxolone (occasional mild gastrointestinal discomfort/hypokalemia, manageable via dose adjustment) [16] (**9**, Figure 2), omaveloxolone (grade 1–2 headache, nausea, reversible transaminase elevation) [17] (**10**, Figure 2), and ibrexafungerp (mild gastrointestinal symptoms, no treatment-related SAEs) [18] (**11**, Figure 2). For HIV inhibitors, bevirimat (**12**, Figure 2, phase I/II) has good tolerability (mild headache/gastrointestinal reactions, no Dose-Limiting Toxicity/SAEs) but development halted due to efficacy limitations [19]; its derivative GSK2838232 (**13**, Figure 2, phase 2a, +cobicistat) shows favorable tolerability and short-term efficacy without safety signals [20]. Optimized candidates GSK3640254 (**14**, Figure 2), and GSK3739937 (**15**, Figure 2), also exhibit mild grade 1–2 gastrointestinal adverse events and no SAEs [21,22]. These results suggest that PTs and their derivatives, featuring mild and reversible Adverse Events, can serve as privileged skeletons for new drug development in medicinal chemistry, with their favorable safety profiles supporting this role.

The broad array of antiviral activities—such as anti-HIV [23], anti-HCV [24,25], anti-IFV [26,27,28], and anti-SARS [29]—exhibited by PTs and their saponins has spurred investigations into PTs derivatives with greater structural diversity. These derivatives demonstrate the potential to further broaden the spectrum of therapeutic applications and optimize clinical outcomes across diverse viral infection contexts. These findings underscore that PTs and their derivatives offer multiple advantages. Notably, PTs and their saponins have demonstrated promising antiviral effects against SARS-CoV-2 and its variants in cell-based assays, which exhibit novel mechanisms of action to form both hydrogen bonds and tight hydrophobic interaction with key targets such as spike (S) protein, 3CLpro, and Mpro, thereby affecting the antiviral potency of the parent molecules. On the other hand, substitutions or modifications at different positions of the PT core structure were found to give rise to diverse PTs derivatives with better antiviral potency. Of significance, GL and other PTs have been proved to simultaneously target two or more pharmacological proteins of SARS-CoV-2 virus and even specific pathways on host cells to exhibit anti-inflammatory effects. Based on its proven efficacy against SARS-CoV and the high structural similarity among coronaviruses, GL shows promising antiviral activity against SARS-CoV-2, with multifaceted mechanisms including binding to the angiotensin-converting enzyme 2 (ACE2) receptor, disrupting the viral membrane, and modulating host immune responses, thus positioning it as a potential therapeutic and prophylactic agent against COVID-19 [30]. UA directly inhibits SARS-CoV-2 proliferation by targeting viral protease and disrupting virus-ACE2 binding. Furthermore, it alleviates COVID-19 complications—such as acute lung injury (ALI) in acute respiratory distress syndrome (ARDS)—by suppressing oxidative stress and modulating inflammatory cytokines [31]. Given that different structural types of PTs provide a rich space for chemical modification and optimization, PTs derivatives have emerged as promising scaffolds for multifunctional anti-SARS-CoV-2 drug development, owing to their capacity to engage with multiple pharmacological targets. This further reinforces the potential of PTs as a crucial privileged framework in designing potent single-molecule anti-SARS-CoV-2 inhibitors with polyvalent characteristics.

Although more than three anti-SARS-CoV-2 drugs have been FDA-approved, effective treatments for certain viral infections remain scarce, and existing therapies either show poor tolerance or limited efficacy. This underscores the need for continuous efforts in designing and developing novel anti-SARS-CoV-2 medications. Therefore, considering the broad-spectrum antiviral activities exhibited by PTs and saponins, many researchers have undertaken the development of a large number of PT derivatives as SARS-CoV-2 inhibitors with diverse targets. To date, and to the best of our knowledge, no review has comprehensively focused on the anti-SARS-CoV-2 activity of PTs. This gap in the literature hinders the efficient translation of PT research into practical drug development. To address this limitation, the present review—which represents, we believe, the first of its kind in this focused area—summarizes recent advances in the antiviral effects and mechanisms of PTs against SARS-CoV-2, providing insights for developing new inhibitors. The literature for this review was collected from multiple scientific databases, including PubMed, Web of Science, and Google Scholar, covering publications from January 2019 to December 2025. The search strategy employed key terms such as pentacyclic triterpenoids, SARS-CoV-2, antiviral, along with relevant compound names (e.g., oleanolic acid, glycyrrhizic acid) and their derivatives. It also examines the structure–activity relationships (SARs) and therapeutic potential of PTs, with a focus on structural modifications at different sites of the PT core. Furthermore, the article evaluates the application prospects of PTs and their saponins in COVID-19 management based on existing evidence, while exploring the rationale for designing multi-target PTs-derived anti-SARS-CoV-2 agents.

## 2. Anti-COVID-19 Potential of PTs by Targeting Key Viral Proteins During SARS-CoV-2 Infection

### 2.1. PTs-Based Entry Inhibitors

**Figure 3:**
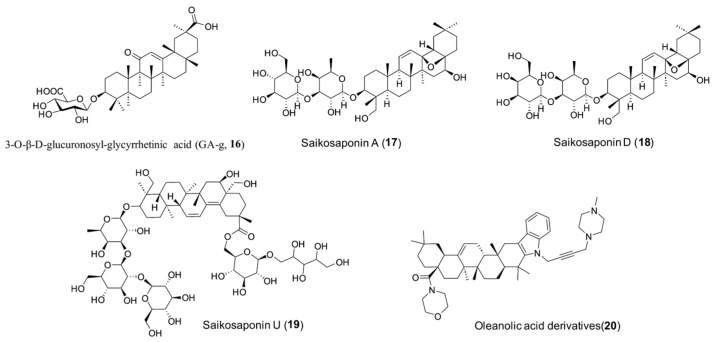
Chemical structures of representative PT saponins **16**–**20**.

The infection mechanism of SARS-CoV-2 and its variants involves multiple steps, including viral attachment, coreceptor binding, and membrane fusion, which are all mediated by the S protein [32,33]. The RBDs within the S1 subunit play a crucial role in viral entry by interacting with the host cell receptor ACE2 [34,35]. Due to their essential function in viral invasion, RBDs have become key targets for antiviral drug development. However, the high mutability of RBDs poses a challenge in identifying broad-spectrum inhibitors. Recent studies have demonstrated that certain PT derivatives can block SARS-CoV-2 entry by targeting the S1 subunit, positioning them as potential viral entry inhibitors [36,37]. For example, Yu et al. [38] reported in 2020 that GL (Table 1), a natural pentacyclic triterpenoid-derived compound, acts as a SARS-CoV-2 entry inhibitor. It disrupts the interaction between the viral RBD and ACE2, with an IC_50_ of 22 µM as confirmed by NanoBit and surface plasmon resonance (SPR) assays. Notably, GL also directly targets the S proteins of MERS-CoV and SARS-CoV, suggesting its potential as a pan-coronaviral candidate. Subsequently, Li et al. [39] found that GL was able to block S-mediated cell attachment to inhibit pSARS-CoV-2 virus into cells, which could bind strongly to the S-RBD with a binding energy of -7.0 kcal/mol based on molecular docking. In continuation with ongoing studies, Yi et al. [40] (2022) confirmed that GA (Table 1) and GA-g (Table 1) (**16**, Figure 3) exhibited remarkably higher affinities with RBD of SARS-CoV-2 relative to GL with an IC_50_ value of 10.9 or 14.1 µM, respectively. It is worth noting that UA and BA (Table 1) were found to also exhibit comparable and even slightly stronger inhibitory potency compared to GA with an IC_50_ value of 9.0 or 15.1 µM [40], respectively, suggesting that diverse types of PTs are tolerated as privileged fragments of SARS-CoV-2 entry inhibitors. The preliminary SARs study indicated that removing the disaccharide fragment attached to the C-3 position of GL led to significantly enhanced inhibitory activity, and its simplification into only one monosaccharide residue was acceptable for inhibitory activity. Consistently, GA could remarkably block SARS-CoV-2 virus in a dose-dependent manner in Vero E6 cells with an IC_50_ value of 3.17 µM [40]. Molecular docking analysis indicated that Y453 is a critical residue mediating GA’s interaction with the spike RBD. Among the tested compounds, GA formed three hydrogen bonds with RBD residues, while GA-g and GL established two and one hydrogen bond(s), respectively. These results illustrate that modifications to GA may pave the way for further research into the rational development of PT-based SARS-CoV-2 inhibitors targeting S-RBD.

Saikosaponin, a member of oleanane saponins, has been previously reported to possess anti-coronavirus efficacy by interfering with early viral replication stages. Numerous studies have shown that saikosaponin A (**17**, Figure 3) exerts anti-inflammatory effects by modulating cytokine and reactive oxygen species (ROS) production as well as lipid metabolism, while saikosaponin D exerts (**18**, Figure 3) antitumor effects by inhibiting cell proliferation and inducing apoptosis and autophagy; additionally, the antiviral mechanisms of saikosaponins (SSs)—especially against SARS-CoV-2—have been partially elucidated [41]. Saurabh et al. [42] revealed that among a series of saikosaponin analogs, saikosaponin U (**19**, Figure 3) exhibits the highest binding affinity for the S protein, as demonstrated by molecular docking analysis. Through computational molecular docking simulations, saikosaponin U was found to fit snugly into the binding pocket formed by multiple amino acids, with a binding energy of −7.27 kcal/mol. This high affinity may be attributed to the presence of an additional oxygen heterocyclic ring, besides the octadehydrene and substituted oxane rings, which likely enables more effective engagement within the extensive binding pocket of the spike glycoprotein. However, the biological activities of these saponins require further investigation to confirm their anti-SARS-CoV-2 potential. More recently, Petrova et al. [43] reported that a set of indolo-oleanolic acid morpholine amide Mannich derivatives (**20**, Figure 3, Table 1) exhibited anti-SARS-CoV-2 pseudovirus activity (IC_50_ = 14.8 μM) and was studied for its interaction with the RBD of the SARS-CoV-2 spike glycoprotein.

**Table 1:** Inhibitory potency of representative PTs against S-RBD and SARS-CoV-2.

Representative PTs	IC_50_	References
Glycyrrhizinic acid (GL, 7)	22 µM against S-RBD	[38]
Glycyrrhetinic acid (GA, 2)	10.9 µM against S-RBD, 3.17 µM against pSARA-CoV-2	[40]
3-O-β-D-glucuronosyl-glycyrrhetinic acid (GA-g, 16)	14.1 µM against S-RBD, 5.37 µM against pSARA-CoV-2	[40]
Ursolic acid (UA, 4)	9.0 µM against S-RBD	[40]
Betulinic acid (BA, 5)	15.1 µM against S-RBD	[40]
Oleanolic acid derivatives (20)	14.8 µM against pSARA-CoV-2	[43]

### 2.2. PT-Based SARS-CoV-2 Fusion Inhibitors

During the life cycle of enveloped viruses, virus–host fusion is mediated by fusion proteins in viral envelopes, such as hemagglutinin (HA2) [44,45] of influenza, E2 of HCV [46], GP of EBOV [47], and GP41 of HIV [48,49]. Substantial evidence indicates that SARS-CoV-2 S2 is crucial in viral entry into host cells, mediating viral-cell membrane fusion. This function positions S2 as a promising target for inhibiting the virus [50,51,52,53]. The interaction between ACE2 and the S1 RBD triggers structural reorganization of the SARS-CoV-2 S2 subunit, transitioning from a prefusion to postfusion conformation. This transformation involves 6-HB formation between HR1 and HR2 domains, enabling viral-cell membrane fusion, which is a critical step for host cell entry [54,55,56,57]. Notably, the S2 subunit exhibits significantly higher sequence conservation compared to the RBD [58]. Thus, the S2 subunit can serve as a conserved and critical target for developing novel pan-viral drugs against Omicron and its variants, by inhibiting the conformational rearrangement of S2 subunits. Although there are currently no FDA-approved SARS-CoV-2 fusion inhibitors, several kinds of small-molecular fusion inhibitors have been reported by various research groups, such as niclosamide [59,60], clofazimine [61,62], salvianolic acid C (Sal-C) [63,64], and salinomycin [65,66]. Notable examples include EK1-peptides [67], a class of HR1-targeting fusion inhibitors currently in phase II trials, which demonstrate broad-spectrum activity against SARS-CoV-2 variants and other coronaviruses. Similarly, Braga et al. [68] reported that niclosamide exhibits potent antiviral effects by inhibiting TMEM16-mediated syncytia formation, supporting its potential COVID-19 therapeutic application.

**Figure 4:**
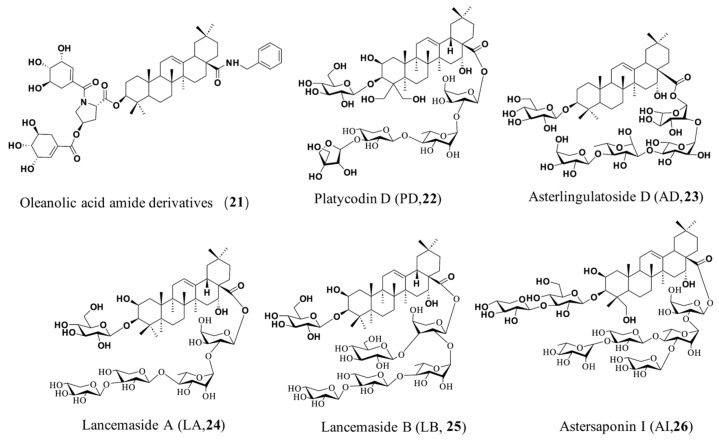
Chemical structures of representative PTs saponins **21**–**26**.

For highly pathogenic enveloped viruses such as HIV, influenza H5N1 virus, and CoVs including SARS-CoV and MERS-CoV, a surrogate pseudovirus (PsV) system assay has become an effective screening method for hit identification of entry inhibitors and for exploration of chemical diversity space in antiviral drug discovery due to its good safety and specificity [69,70]. To date, several classes of natural SARS-CoV-2 entry inhibitors bearing the PT skeleton have been reported. Cao et al. [71] demonstrated in their research that oleanonic acid was identified as a novel natural SARS-CoV-2 entry inhibitor, with potent antiviral activity against Alpha and Beta variants at the micromolar level. In cell-based assays, it exhibited an IC_50_ of 1.4 µM and a favorable selectivity index (SI = 39), highlighting its potential as a promising therapeutic candidate. Further investigations show that oleanonic acid inhibits SARS-CoV-2 entry by blocking membrane fusion, without affecting RBD binding. Our study identified a series of oleanolic acid amide derivatives as promising broad-spectrum SARS-CoV-2 entry inhibitors. The representative compound (**21**, Figure 4, Table 2), which features an N, O-disubstituted butterfly-shaped *L*-hydroxyproline scaffold at the C-3 position of OA, exhibited high binding affinity for the Omicron spike protein (K_D_ = 5.36 µM) and potently inhibited the entry of pseudotyped SARS-CoV-2, Delta, and Omicron viruses (IC_50_ = 2.86–8.13 µM). Notably, compound **21** also showed potent activity against live Omicron virus in cellular models, with superior efficacy in a pre-treatment model (IC_50_ = 1.08 µM) versus a full-time treatment model (IC_50_ = 5.79 µM), indicating an early-stage mechanism. Mechanistic studies revealed that compound **21** does not block S1-ACE2 attachment but specifically inhibits S2-mediated membrane fusion. These findings establish compound **21** and its analogs as a novel class of viral fusion inhibitors targeting a critical step in Omicron entry [72].

Oleanane-type PT saponins from plant sources, on the other hand, also act as novel SARS-CoV-2 fusion inhibitors, which can target multiple stages of viral and cellular membrane fusion through different mechanisms of antiviral action. Othman et al. [73] isolated Oleanane-type PT saponins from the halophyte Anabasis setifera, which exhibited anti-SARS-CoV-2 activity with IC_50_ values ranging between 2.339 and 6.837 µg/mL. Kim et al. [74] identified platycodin D (PD, **22**, Figure 4, Table 2), a major pentacyclic triterpenoid saponin in *Platycodon grandiflorum* (PG), as a dual-pathway inhibitor of SARS-CoV-2 entry, showing micromolar potency against both lysosomal and TMPRSS2-mediated infection routes. This inhibition occurs by redistributing membrane cholesterol to prevent viral-cell membrane fusion, presenting a promising novel therapeutic strategy against SARS-CoV-2 infection. Interestingly, the natural PT saponin asterlingulatoside D (**23**, Figure 4, Table 2)—originally isolated from *Aster lingulatus* whole plants—was shown to exhibit more potent inhibitory activity against SARS-CoV-2 entry, with an IC_50_ of 0.64 µM (PD, IC_50_ = 1.34 µM) [75]. The cell fusion assay demonstrated that asterlingulatoside D inhibits SARS-CoV-2 entry into host cells by blocking viral-host membrane fusion. The derivative of asterlingulatoside D exhibited broad-spectrum inhibition against multiple SARS-CoV-2 variants (Alpha, Beta, Gamma, Delta, Omicron) in Vero cells with micromolar efficacy, suggesting PT saponins’ potential against emerging variants [75]. Kim et al. [76] identified lancemasides A (LA, **24**, Figure 4, Table 2) and B (LB, **25**, Figure 4, Table 2) from *Codonopsis lanceolata* as potent SARS-CoV-2 entry inhibitors, exhibiting IC_50_ values of 1.77 µM and 4.97 µM, respectively, in a pseudovirus assay. These oleanane-type saponins, featuring a C-3 β-D-glucuronic acid moiety, exhibited comparable efficacy to platycodin D against multiple variants in pseudovirus assays. Their mechanism likely involves cholesterol modulation and membrane fusion inhibition, mirroring PD’s activity. Kim et al. [77] reported that Astersaponin I (AI, **26**, Figure 4, Table 2)—a natural viral fusion inhibitor isolated from *Aster koraiensis*—bears a disaccharide chain at the C-3 position of its aglycone. In vitro studies showed that AI inhibits infection by pseudotyped SARS-CoV-2 variants and their ancestral virus, with comparable IC_50_ values (1.91–2.04 μM). The researchers proposed a mechanism whereby AI disrupts viral-cell membrane fusion by modulating membrane cholesterol and protruding sugar moieties at the aglycone’s C-28 position upon plasma membrane incorporation, thereby preventing SARS-CoV-2 infection. Collectively, preliminary SAR analyses indicated that the overall conformation of these natural pentacyclic triterpenoid (PT) saponins is critical for antiviral activity. The oleanane-type triterpene hydrophobic backbone, along with bound hydrophilic sugar moieties—such as β-monosaccharide or disaccharide units at the C-3 position or oligosaccharides at the C-28 position—are structurally indispensable for inhibitory potency against membrane fusion.

To clarify the importance of sugar moieties at the C-3 or C-28 position and identify critical oxidations in the triterpenoid backbone of PD that contribute to its therapeutic effects, Jang et al. [75] designed and synthesized a series of PD derivatives. The SARs of saponin-based antiviral agents against SARS-CoV-2 virus revealed that the β-D-glucose at the C-3 position, the oligosaccharide residue at the C-28 position that is composed of (→3)-β-D-Xyl-(1→4)-α-L-Rha-(1→2)-β-D-Ara-(1→) as the last three sugar units, and the hydroxyl group at the C-16 position of the aglycone are vital components of PT saponin-based SARS-CoV-2 fusion inhibitors. Among the synthesized saponins, the asterlingulatoside D derivative bearing an additional D-xylose moiety at the C-28 oligosaccharide exhibited a similar inhibitory potency as asterlingulatoside D (IC_50_ = 0.64 μM) and about 2-fold increased inhibition against pSARS-CoV-2 compared to PD. Further investigations revealed that the synthetic PT saponins effectively inhibit membrane fusion across multiple SARS-CoV-2 variants, which is a critical step shared by both endosomal and TMPRSS2-mediated viral entry pathways. This broad-spectrum activity highlights their potential as pan-coronavirus fusion inhibitors. The findings establish a foundation for developing optimized saponin-based therapeutics derived from PD, offering a promising approach not only against SARS-CoV-2 but also for future emerging coronaviruses.

**Table 2:** Inhibitory activity against pSARS-CoV-2 entry of representative PTs saponins.

Representative PTs Saponins	IC_50_	References
Oleanonic acid (OA, 1)	1.4 µM	[71]
Oleanolic acid amide derivatives (21)	2.8–8.13 µM (SARS-CoV-2, Delta, and Omicron viruses)	[72]
Platycodin D (PD, 22)	1.34 µM	[75]
Asterlingulatoside D (23)	0.64 µM	[75]
Lancemasides A (LA, 24)	1.77 µM	[76]
Lancemasides B (LB, 25)	4.97 µM	[76]
Astersaponin I (AI, 26)	1.91–2.04 µM (SARS-CoV-2 and its variants)	[77]

**Figure 5:**
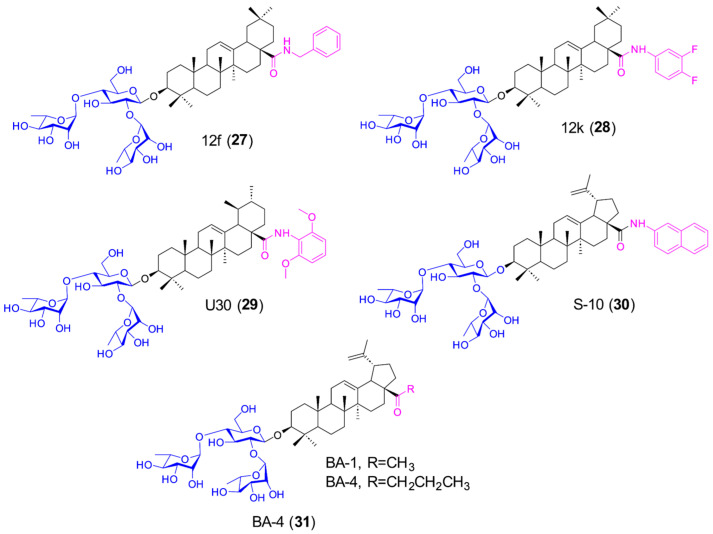
Chemical structures of PT saponins **27**–**31** as SARS-CoV-2 fusion inhibitors. Blue indicates the trisaccharide side chain, and red indicates the key substituent.

To identify potent PT saponin-derived SARS-CoV-2 fusion inhibitors, our group employed an S protein pseudovirus system to screen a semisynthetic PT saponin library, leading to the discovery of OA benzyl ester saponins as novel viral entry blockers. The chacotriose residue (α-L-rhamnopyranosyl-(1→2)-[α-L-rhamnopyranosyl-(1→4)]-β-D-glucopyranosyl) in these PT saponins has been a major focus of SAR research. Key modifications explored include the removal or repositioning of its sugar units, conjugation with different aglycones such as UA or BA, and the attachment of hydrophobic chains at the C-28 position through ester, amide, or ether linkers. Among the synthesized 3-O-β-chacotriosyl PT derivatives, compounds 12f (**27**, Figure 5, Table 3) and 12k (**28**, Figure 5, Table 3) [78] featuring C-17 amide linkers exhibited potent anti-SARS-CoV-2 activity, showing IC_50_ values of 4.37 μM and 5.68 μM with selectivity indices >23 and >18, respectively. Notably, the structurally related UA derivative UA-30 (**29**, Figure 5, Table 3) [79] demonstrated broad-spectrum inhibition against multiple variants (Alpha–Omicron), with IC_50_ values of 0.04–11.57 µM in pseudovirus assays. Additionally, our medicinal chemistry efforts showed the 3-O-β-chacotriosyl BA amide derivative S-10 [80] (**30**, Figure 5, Table 3) also demonstrated broad antiviral activity (IC_50_ = 0.82–5.45 μM) against Omicron and related variants, which showed appreciable pharmacokinetic properties in vitro. The 3-O-β-chacotriosyl PT amide derivatives specifically inhibited SARS-CoV-2 variant (including Omicron) spike-mediated entry, as demonstrated through dose-dependent pseudovirus (SARS-CoV-2 S/HIV-luciferase) and SPR assays.

**Table 3:** Inhibitory effects of representative PT saponins on the entry of SARS-CoV-2.

Representative PTs Saponins	IC_50_	References
12f (27)	4.37 µM against pSARA-CoV-2	[78]
12k (28)	5.68 µM against pSARA-CoV-2	[78]
UA-30 (29)	Pseudovirus entry assay (Alpha, Beta, Gamma, Delta, Omicron pseudotypes): IC_50_ = 0.04–11.57 μM	[79]
S-10 (30)	Pseudovirus entry assay (Alpha, Beta, Gamma, Delta, Omicron pseudotypes): IC_50_ = 0.82–5.45 µM	[80]
BA-4 (31)	Pseudovirus entry assay (Alpha, Beta, Gamma, Delta, Omicron pseudotypes): IC_50_ = 2.73–5.19 µM	[81]

**Figure 6:**
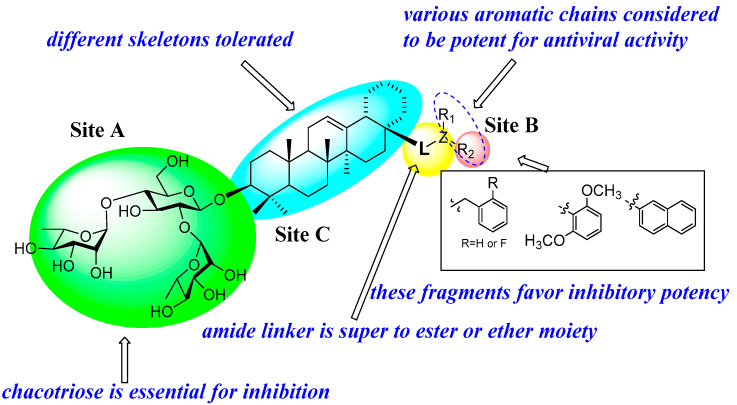
SARs of 3-O-β-chacotriosyl PT saponins derivatives.

As shown in Figure 6, a SAR study was also performed [78,79,80], which illustrated that the chacotriose moiety is essential for inhibition, and different styles of aglycone may be tolerated, with the substituent at the C-28 position playing a critical role in potency. Moreover, the SAR study concluded that the presence of an amide linker at the C-17 position of the PT aglycone has been considered to be potent for inhibitory activity, of which replacement with ester moiety as its bioisostere can lead to increase in cytotoxicity, thus reducing selectivity index. Further SAR analysis revealed that substitution of the C-17 carboxyl group with a hydroxyl group led to a complete loss of inhibitory activity. In contrast, the benzyl ether derivative of the hydroxyl group exhibited significant inhibitory potency against pseudotyped SARS-CoV-2 entry compared to its benzyl amide analog. These findings suggest that potency enhancement correlates with the lipophilicity of C-17 substituents in PTs. SAR studies showed that introducing a chlorine atom at the C-28 position of the PT core structure enhanced inhibitory potency against pseudotyped SARS-CoV-2 entry. However, this modification caused high cytotoxicity, leading to poor SI. Structural analysis indicates that the C-28 substituent properties (type, size) significantly influence PTs’ anti-pSARS-CoV-2 activity, with optimal inhibition observed for either short linear chains (1–4 atoms) or cyclized aliphatic/aromatic groups (particularly benzyl, o-chlorophenyl, or naphthyl), underscoring the crucial role of C-17 hydrophobicity in maintaining potent antiviral effects against pseudotyped SARS-CoV-2. Building on these modifications, 3-O-β-chacotriosyl PT amide derivatives can be synthesized to gain insights for developing novel SARS-CoV-2 entry inhibitors with potent antiviral activity.

**Figure 7:**
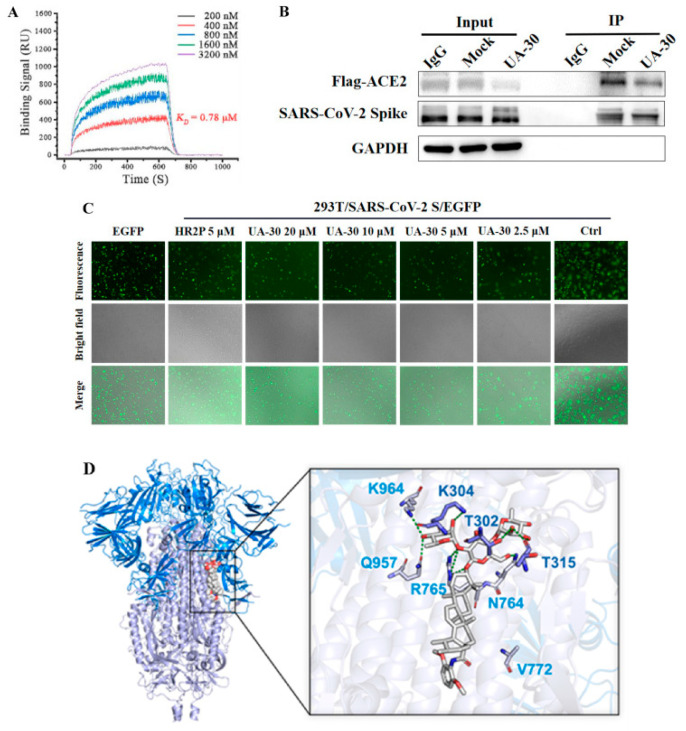
(**A**) SPR analysis of the interaction between UA-30 with SARS-CoV-2 S-trimer. (**B**) The binding of SARS-CoV-2 S protein and ACE2 (Anti-Flag) in the presence or absence of UA-30 (40 μM) was detected by Co-IP assays. IgG was included as a negative control. (**C**) Green fluorescence labels HEK-293T effector cells transfected with the SARS-CoV-2 spike protein or the control vector. The dose-dependent inhibitory effect of UA-30 on SARS-CoV-2 S mediated cell–cell fusion. (**D**) Docking analysis of compound U-30 with S protein (7TF8). (**A**–**C**) were adapted from Reference [79], which is made available under Elsevier’s COVID-19 resource center permissions for unrestricted research reuse.

Figure 7 illustrates the antiviral mechanism of UA-30 [79], showing that it appears to block S-mediated membrane fusion at the prefusion stage by targeting the S2 subunit. This activity was validated by SPR analysis, Co-Immunoprecipitation (Co-IP) assay, cell–cell fusion assay, and circular dichroism (CD) spectroscopy, which collectively demonstrate that UA-30 prevents viral entry into host cells [79]. The 3-O-β-chacotriosyl PT amide derivatives inhibit SARS-CoV-2 through targeted binding at the S1/S2 subunit interface, stabilizing the spike protein’s prefusion conformation. Molecular docking and mutational studies demonstrate how the chacotriose moiety embeds within the S2 cavity, forming critical hydrogen bonds with Thr302, Lys304, Thr315, Asn764, Arg765, Gln957 and Lys964 to prevent membrane fusion. These structural interactions, particularly evident in compound U-30 (Figure 7D), provide a molecular basis for developing this saponin class as effective fusion inhibitors against SARS-CoV-2.

BA derivatives have also demonstrated considerable anti-SARS-CoV-2 activity. Liu et al. reported that these compounds effectively inhibit the entry of the Omicron variant into host cells. Among them, the BA amide derivative S-10 [80] exhibited broad-spectrum antiviral potency against Omicron and other variants, along with favorable pharmacokinetic properties. Similarly, BA saponin derivative BA-4 [81] (**31**, Figure 5, Table 3) showed inhibitory activity against pseudotyped Omicron and other variants, with IC_50_ values between 2.73 and 5.19 μM. Further mechanistic investigations—including pSARS-CoV-2 assays, SPR analysis, Co-IP, cell–cell fusion assays, molecular docking, and mutagenesis studies—revealed that BA-4 stabilizes the S protein in its prefusion conformation, thereby interfering with membrane fusion and inhibiting viral entry.

### 2.3. PTs-Based SARS-CoV-2 3CLpro Inhibitors

The SARS-CoV-2 3CL protease (3CLpro, Mpro) plays an essential role in viral replication by processing polyproteins pp1a and pp1b to generate 16 functional non-structural proteins (Nsp1-16) [82,83]. Many of these are components of viral replication and transcription complexes. For example, pp1a and pp1b must be cleaved by 3CLpro to release the Nsp protein, and the released Nsp, such as Nsp12 (RdRp) and Nsp14 (proofreading enzyme), are required for viral transcription and replication [84,85,86]. Without the function of 3CLpro, the virus cannot complete the replication and transcription steps. Blocking the function of 3CLpro can block the life cycle of the virus. Furthermore, 3CLpro exhibits high structural and sequence conservation in SARS-CoV-2 [87], limiting viral escape through mutation. This feature highlights its potential as a promising antiviral target.

**Figure 8:**
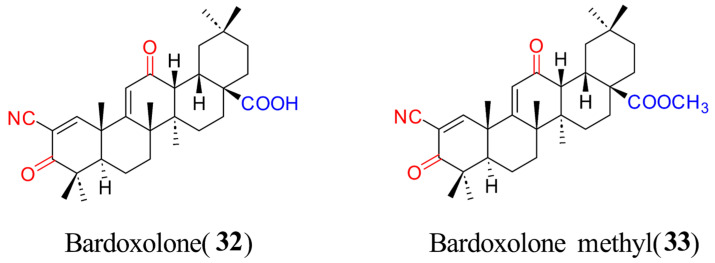
Chemical structures of SARS-CoV-2 3CLPro inhibitors **32** and **33**.

Recently, Sun et al. [88] reported that bardoxolone (**32**, Figure 8, Table 4) and bardoxolone methyl (**33**, Figure 8, Table 4) belonging to members of OA derivatives, two Nrf2 activators in clinical trials, could inhibit 3CLpro enzyme activity with an IC_50_ value of 27.99 μM or 5.81 μM, respectively, thereby blocking SARS-CoV-2 replication in a cell-based assay with an IC_50_ value of 0.43 μM or 0.29 μM, respectively. Preliminary SAR analysis revealed that C-17 methyl esterification in the PT core structure enhances inhibitory potency against both 3CLpro and the virus, though it also increases cytotoxicity. Further investigation revealed that bardoxolone could bind SARS-CoV-2 Mpro in a reversible covalent manner [88]. Interestingly, molecular docking reveals that bardoxolone methyl and bardoxolone bind at the Mpro domain I-II interface, forming hydrogen bonds with Arg40 and hydrophobic contacts with Phe181/Val186, while potentially establishing a covalent interaction with Cys85 [84]. Moreover, the methyl fragment at the C-28 position of bardoxolone methyl may neutralize the negative charge of bardoxolone, which is helpful in decreasing its electrostatic repulsion with Glu55 residue to improve its binding affinity with 3CLpro.

**Table 4:** Antiviral activities of SARS-CoV-2 3CLPro inhibitors (**32** and **33**) in Vero cells.

SARS-CoV-2 3CLPro Inhibitors	IC_50_	References
Bardoxolone (**32**)	22.99 µM against 3CLPro, 0.43 µM in Vero cells	[88]
Bardoxolone methyl (**33**)	5.81 µM against 3CLPro, 0.29 µM in Vero cells	[88]

**Figure 9:**
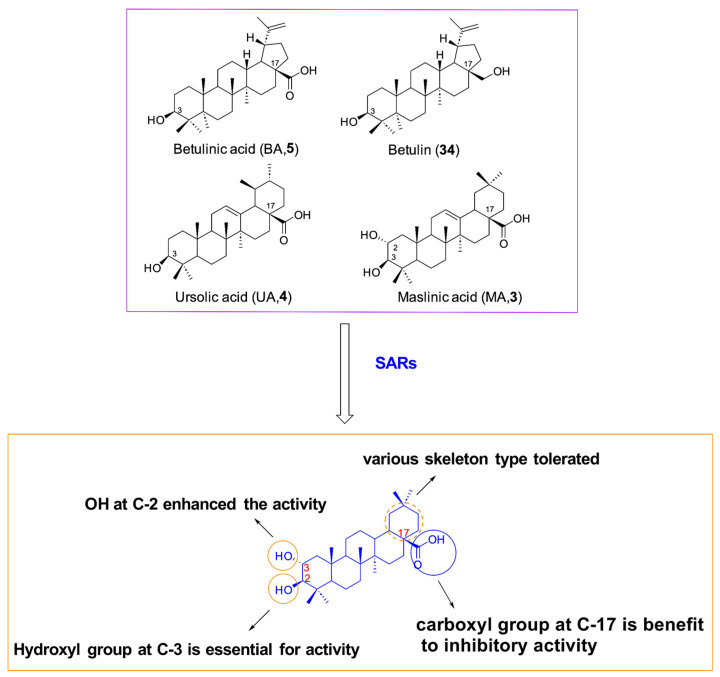
SARs of natural PTs as SARS-CoV-2 3CLPro inhibitors.

In continuation with the above research findings, Alhadrami and his team [89] found that BA, UA, and MA (Table 5) exhibited promising inhibition against SARS-CoV-2 Mpro protein with IC_50_ values of 10.0 µM, 12.57 µM, 3.22 µM, respectively, while betulin (**34**, Figure 9, Table 5) has much lower activity (IC_50_ = 89.67 µM). The SAR analysis demonstrated that C-2 and C-3 hydroxyl groups of the steroidal scaffold are critical for antiviral activity, forming essential hydrogen bonds with 3CLpro’s Thr25 and Ser46 residues. BA was found to form a stable hydrogen bond with His41 of 3CLpro, while MA could form more and stronger hydrogen bonds with His41, Ser46 residues based on molecular docking studies [89], which is consistent with the data on inhibitory enzymes. At the same time, Sand’s team [90] demonstrated GL could inhibit SARS-CoV-2 3CLpro with an IC_50_ value of 30 µM, thereby effectively blocking virus replication. These findings indicate that C-3 modifications of PTs could facilitate the development of novel PT derivatives with improved potency and SI, provided that glycosylation at this position is avoided.

**Figure 10:**
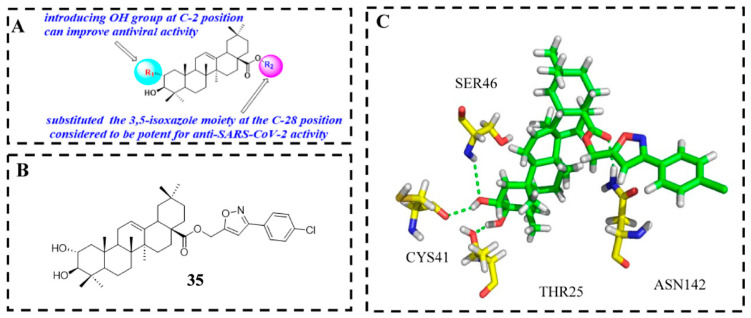
Antiviral mechanism of compound **35**. (**A**) SARs of OA and MA derivatives as SARS-CoV-2 3CLPro inhibitors; (**B**) chemical structure of compound **35**; (**C**) docking data of **35** with Mpro (6LU7), In the figure, blue bonds represent N, gray bonds represent H, red bonds represent O, green bonds represent C, and light green dashed lines represent hydrogen bonds.

In addition, Soltane et al. [91] synthesized a variety of OA and MA derivatives bearing isoxazole at the C-28 position as novel SARS-CoV-2 Mpro inhibitors. The most active synthesized MA derivative was found to be the compound (**35**, Figure 10B), namely (3-(4-chlorophenyl) isoxazol-5-yl) methyl-(2α, 3β)-2, 3-dihydroxyolean-12-en-28-oate, which exhibited an IC_50_ value of 4.12 μM against wild SARS-CoV-2 with a satisfactory SI (≥7.5). Moreover compound **35** also displayed promising anti-MERS-CoV activity (IC_50_ = 6.25 μM) at non-toxic concentrations and strong inhibition against NRCE-HKU270 virus at micromolar concentrations [91], signifying that it has potential broad-spectrum anti-coronavirus activity. The SAR analysis revealed that 2- and 3-position hydroxyl groups on the steroidal core are essential for antiviral activity, forming key hydrogen bonds with Mpro’s Thr25 and Ser46 residues. On the other hand, a SAR study revealed that incorporation of the 3, 5-isoxazole side chain containing a *p*-chlorophenyl substitution at the C-28 position of MA has been considered to be potent for anti-SARS-CoV-2 activity. Docking analysis revealed derivative **35**_’_s optimal positioning within Mpro’s branched pocket, forming key hydrogen bonds with Thr25, Ser46, Cys41, and Asn142 [91]. Further mechanism of action studies demonstrated that **35** efficiently impairs viral replication and adsorption despite its virucidal effect, signifying that **35** as a potential dual-target inhibitor can target both SARS-CoV-2 S and 3CLpro proteins.

### 2.4. PTs-Based SARS-CoV-2 nsp Inhibitors

**Figure 11:**
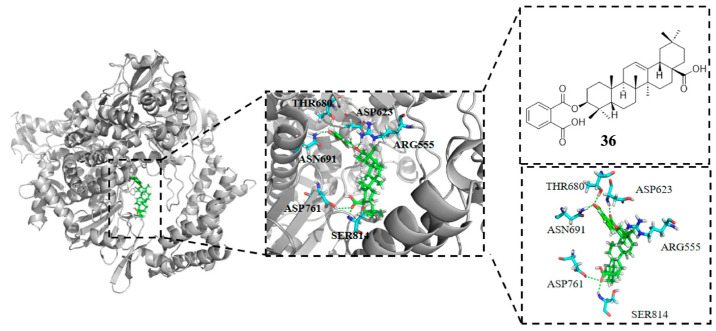
Chemical structure of **36** and docking data of **36** with SARS-CoV-2 RdRp (7bve).

RNA-dependent RNA polymerase (RdRp, nsp12) is a core component of the SARS-CoV-2 viral replication and transcription complex [92], which produces viral mRNA through a discontinuous transcription mechanism to encode viral proteins, and interacts with nsp7, nsp8, and nsp14 to form an activity-enhancing complexes to ensure efficient RNA synthesis while reducing the error rate during replication [93]. Given their high conservation, SARS-CoV-2 nsp family members like RdRp and nsp7 serve as primary targets for antiviral drug development [94]. The research discussed in this subsection is based on computational approaches (such as molecular docking and in silico ADME predictions). Sharma et al. [95] reported that certain C-3-substituted ester derivatives of OA, such as 3-phthaloyl OA (**36**, Figure 11), exhibited strong binding affinity to RdRp (7BVE). In their molecular docking studies, the derivative achieved a MolDock score of –118.28 kcal/mol in silico, illustrating that PTs derivatives show potential for inhibiting SARS-CoV-2 RdRp to a certain extent. As depicted in Figure 11, compound **36** can form multiple hydrogen bonds with Ser841, Asp761, Asn691, Thr680, Arg555, Asp623 residues to stabilize the combination with RdRp, highlighting the importance of introducing phthalate esters at the C-3 position of OA.

**Figure 12:**
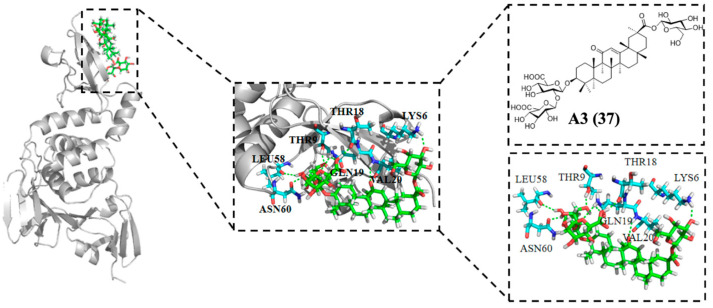
Chemical structure of compound **37** and docking data of **37** with SARS-CoV-2 nsp7 (7JIT).

Interestingly, GL was found to occupy the binding pocket located between the NiRAN domain and β-hairpin structure of RdRp based on molecular docking, which may interfere with the polymerase activity [96]. However, additional in vitro and in vivo experiments are needed to validate the anti-SARS-CoV-2 activity of PTs, as current biological data remain limited. Yi et al. [40] reported that Licorice-saponin A3 (**37**, Figure 12)—a β-D-glucopyranosyl glycoside derivative of GL—potently inhibited SARS-CoV-2 infection in vitro, with an IC_50_ of 75 nM. Further studies revealed that A3 could strongly bind to nsp7 and also target S-RBD [96], suggesting that A3 can be regarded as a double-target SARS-CoV-2 inhibitor. Thus, A3 blocks SARS-CoV-2 infection by targeting both viral entry and replication. As shown in Figure 12, A3 can form important hydrogen bonds with Leu58, Thr9, Asn60, Gln19, Thr18, Val20, and Lys16 residues, respectively. Interestingly, GL can only target S protein without binding with nsp7, which is different from A3. These results demonstrate that modification of GL not only can improve antiviral activity but also have an important effect on their mechanism of action, which provides an opportunity for designing novel dual-target anti-SARS-CoV-2 inhibitors. Kumar et al. [97] reported that UA stably bound to SARS-CoV-2 nsp15, potentially disrupting viral replication as indicated by molecular docking analysis. However, in vitro and in vivo validation is needed to confirm these findings. Molecular docking demonstrated that UA could make multiple strong hydrogen bonds with Lys290, Gly248, Thr341, His250 and His235 residues in nsp15, and this is in agreement with the data of molecular dynamics (MD) studies. This result suggests that UA is a promising nsp15 inhibitor that can be considered as a promising lead phytochemical.

### 2.5. PTs-Based SARS-CoV-2 PLpro Inhibitors

PLpro has been shown to cleave polyproteins, deubiquitinate, and interfere with host immunity, making it a key protein in the virus replication process and for immunity evasion [98,99]. Some studies have confirmed that PTs have good binding ability to bind PLpro based on molecular docking. For example, Rudrapal et al. [100] demonstrated that saikosaponin A and betulinic acid exhibited strong binding affinity to PLpro via molecular docking, MD simulations, and ADMET analyses. These findings provide novel leads for identifying potential anti-COVID-19 drug candidates. Docking studies revealed distinct binding patterns: saikosaponin A formed hydrogen bonds with PLpro’s Asn109, Tyr112, Gln269, and Tyr273, while betulinic acid lacked such interactions. Computational analyses further predicted ursolic acid’s binding to catalytic residues His273 and Asp287, suggesting PLpro inhibitory potential. These results support further hit-to-lead optimization for anti-COVID-19 drug discovery, pending biological validation. Collectively, these findings provide a theoretical foundation for developing PTs-based SARS-CoV-2 PLpro inhibitors in subsequent biological research.

## 3. Anti-Inflammatory Pathways

**Figure 13:**
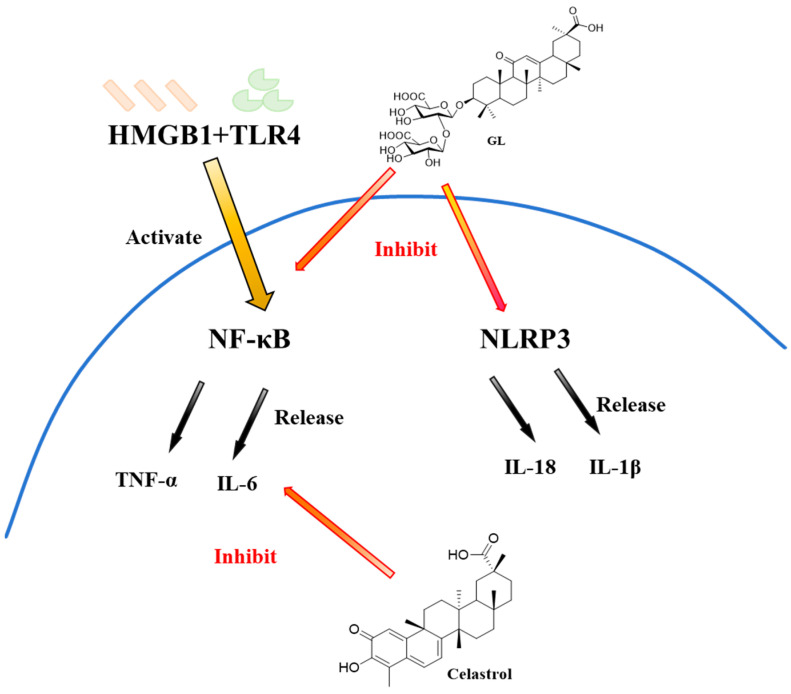
GL and celastrol showing anti-inflammatory ability.

Clinical evidence strongly associates COVID-19 severity with elevated pro-inflammatory cytokines, indicating cytokine storm involvement in critical cases [101]. As shown in Figure 13, GL suppresses this inflammatory cascade by inhibiting TLR4-mediated NF-κB activation, thereby reducing production of IL-6, TNF-α and other cytokines linked to pulmonary damage and respiratory failure [102]. Therefore, the inhibition of TLR4 by GL is of great significance for alleviating cytokine storm and protecting lung tissue. Gowda et al. [103] further demonstrated that GL exhibits dual activity: it blocks viral entry into host cells by targeting the RBD, while suppressing pro-inflammatory mediators through direct binding to HMGB1, making it a key extracellular inflammation mediator that stimulates IL-1β and ferritin production, exacerbating SARS-CoV-2 infection severity. GL exerts anti-inflammatory effects by suppressing HMGB1 release from macrophages, thereby disrupting HMGB1-IL-1β and ferritin-IL-1β crosstalk and preventing macrophage hyperactivation.

Fuzo et al. [104] reported in 2022 that celastrol not only disrupted SARS-CoV-2 and variant replication but also reduced IL-6 secretion, which is a cytokine pivotal for host immune and inflammatory regulation. These findings indicate celastrol’s potential as an anti-SARS-CoV-2 agent, attributed to its dual anti-inflammatory and antiviral activities. Collectively, these results highlight that PT-based inhibitors with combined antiviral and anti-inflammatory potency may represent a promising therapeutic strategy for SARS-CoV-2 and variant infections.

## 4. Development of PT-Based SARS-CoV-2 Inhibitors as Antiviral Drugs

The design of multi-target drugs, which serves as a valid approach for synergistic inhibition of multiple pathways, can both enhance therapeutic efficacy and mitigate resistance risks. Supporting this concept, clinical evidence has demonstrated the benefit of multi-target activity. In a trial by Xu et al. [105], patients receiving the Glycyrrhizin-containing granule—a compound with known multi-target anti-inflammatory and antiviral properties—exhibited more rapid fever resolution and a shorter duration of severe symptoms compared to standard care. The treatment also decreased the requirement for mechanical ventilation and intensive care, underscoring its role in improving outcomes against SARS-CoV-2 infection. At present, the development of multi-target agents—particularly dual-target compounds—for antiviral therapy is gaining traction as a strategy to address drug resistance [106,107]. Given the potential for resistance to FDA-approved anti-SARS-CoV-2 drugs and the ongoing mutation of the SARS-CoV-2 virus, dual or even multi-target inhibition has emerged as a promising strategy for COVID-19 therapy.

**Figure 14:**
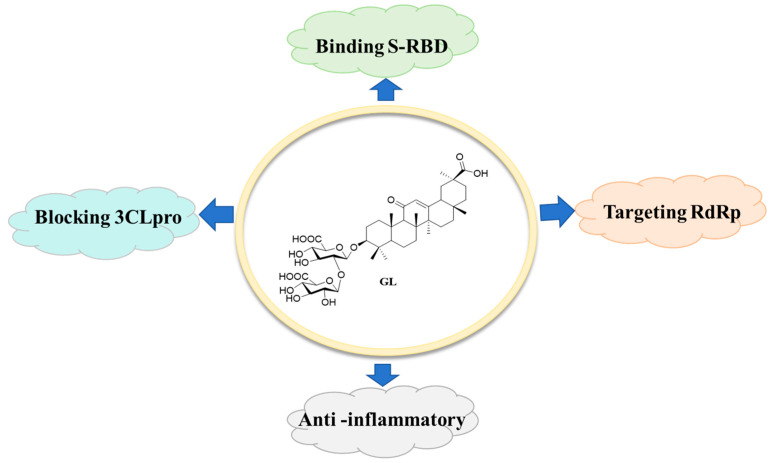
GL can block SARS-CoV-2 bearing the multi-target feature.

PTs and their saponins exhibit broad-spectrum anti-SARS-CoV-2 effects through dual mechanisms of viral entry inhibition and replication suppression. By simultaneously targeting S protein and Mpro along with other viral components, these compounds demonstrate significant potential as multi-target therapeutic candidates against COVID-19. As presented in Figure 14, GL is a clinical drug, and its effects against SARS-CoV-2 have been shown to directly affect S-RBD, 3CLpro, and RdRp proteins, suggesting that GL can serve as a privileged scaffold to develop anti-SARS-CoV-2 drugs with multiple targets. Notably, GL not only exhibits direct antiviral activity against SARS-CoV-2, but also displays significant anti-inflammatory effects, further supporting its potential for a “multi-target” feature by affecting both viral proteins and multiple pathways in the host cells [93]. Thus, GL, as a potential multi-target inhibitor, warrants further evaluation in both animal and clinical trials for COVID-19 treatment. On the other hand, compound A3 [40], a GL derivative, also possesses dual-target feature targeting S-RBD and nsp7 proteins of SAS-CoV-2, highlighting that modification of PTs is helpful in providing a new strategy for obtaining novel anti-SARS-CoV-2 drugs with multi-target feature. Besides GL and its analogs, other natural PTs such as BA and UA have been found to simultaneously target S, Mpro, and other proteins of SARS-CoV-2 to display potential antiviral activity, further highlighting that PTs can serve as privileged scaffolds to develop potential dual-target and even multi-target anti-SARS-CoV-2 inhibitors.

Despite their unique antiviral mechanism and promising efficacy, PT-based anti-SARS-CoV-2 candidates face critical pharmaceutical challenges including dose-limiting solubility, poor intestinal permeability, and inadequate oral bioavailability [108,109]. Current strategies focus on both structural optimization through medicinal chemistry approaches to enhance biopharmaceutical properties, and advanced formulation development utilizing nanocarriers (nanoemulsions, solid lipid nanoparticles) to improve pharmacokinetic profiles and overall druggability [110,111,112].

## 5. Conclusions and Future Perspectives

Pentacyclic triterpenes and their derivatives have emerged as promising candidates for antiviral research, particularly against coronaviruses like SARS-CoV-2. Given the persistent threat posed by evolving coronaviruses to global health and economies, developing novel therapeutics targeting multiple viral components remains crucial. These natural scaffolds offer potential for creating next-generation anti-SARS-CoV-2 drugs with improved efficacy against variants.

This review, based on a comprehensive analysis of literature from 2019 to date, summarizes current progress and highlights the emerging role of PT-based SARS-CoV-2 inhibitors within antiviral drug discovery. In addition to GL, many PTs such as OA, UA, BA, and their saponins also show notable in vitro antiviral potential against SARS-CoV-2. The inhibitory effect of PTs on SARS-CoV-2 is suggested to be achieved through multi-target antiviral mechanism, which may be particularly relevant in the context of high mutation frequency in the viral spike protein. In addition, PTs also possess significant anti-inflammatory effects, and this dual activity could further support their potential utility in viral suppression and host protection.

Current antiviral development increasingly focuses on compounds with multiple mechanisms of action to overcome resistance challenges. Evidence indicates that certain PTs can simultaneously engage both spike proteins and viral proteases, suggesting their potential as scaffolds for developing broad-spectrum antiviral agents. Despite these promising in vitro observations, significant questions remain unanswered. Limited data exist regarding the in vivo efficacy and pharmacokinetic behavior of most PT derivatives. The translation of in vitro antiviral activity to clinical settings requires substantial investigation into bioavailability, metabolic stability, and tissue distribution. Furthermore, comprehensive structure–activity relationship studies are needed to guide rational optimization of these compounds.

Priority research directions should include advanced structural modification approaches to enhance potency while maintaining favorable drug-like properties; detailed mechanistic studies to elucidate interactions with both viral targets and host factors; development of formulations that improve solubility and metabolic stability; and rigorous assessment in physiologically relevant models, including animal infection systems.

In summary, pentacyclic triterpenoids represent a promising starting point for antiviral discovery efforts. Through continued investigation of their unique chemical and biological properties, these natural products could potentially contribute to therapeutic strategies addressing current and future coronavirus threats.

## Figures and Tables

**Table 5:** Antiviral activities of representative PTs as inhibitors of SARS-CoV-2 3CLpro.

Representative PTs	IC_50_	References
Betulinic acid (BA, **5**)	10 µM against 3CLpro	[89]
Betulin (**34**)	89.67 µM against 3CLpro	[89]
Ursolic acid (UA, **4**)	12.57 µM against 3CLpro	[89]
Maslinic acid (MA, **3**)	3.22 µM against 3CLpro	[89]

## Data Availability

No new data were created or analyzed in this study. Data sharing is not applicable to this article.

## Abbreviations

The following abbreviations are used in this manuscript:

CoVs	Coronaviruses
SARS-CoV-2	Severe acute respiratory syndrome coronavirus 2
RdRp	RNA-dependent RNA polymerase
NPs	Natural products
PT	Pentacyclic triterpenoid
OA	Oleanolic acid
GA	Glycyrrhetinic acid
MA	Maslinic acid
UA	Ursolic acid
BA	Betulinic acid
GL	Glycyrrhizinic acid
SAEs	Severe adverse events
S	Spike
ACE2	Angiotensin-converting enzyme 2
ALI	Acute lung injury
ARDS	Acute respiratory distress syndrome
SARs	Structure–activity relationships
RBDs	Receptor-binding domains
SPR	Surface plasmon resonance
ROS	Reactive oxygen species
SSs	Saikosaponins
Sal-C	Salvianolic acid C
PsV	Pseudovirus
PG	Platycodon grandiflorum
LA	Lancemasides A
AI	Astersaponin I
platycodin D	PD
Co-IP	Co-Immunoprecipitation
CD	Circular dichroism
3CLpro	3CL protease
Nsp	Non-structural proteins
MD	Molecular dynamics
